# Supplementary material for: IntegronFinder 2.0: Identification and Analysis of Integrons across Bacteria, with a Focus on Antibiotic Resistance in Klebsiella
Source: Microorganisms. 2022 Mar 24;10(4):700. doi: 10.3390/microorganisms10040700 (PMC9024848; doi:10.3390/microorganisms10040700)
Supplement: Supplementary file 1 [file microorganisms-10-00700-s001.zip › microorganisms-1643943-supplementary.pdf]

microorganisms

MDPI

Supplementary Material

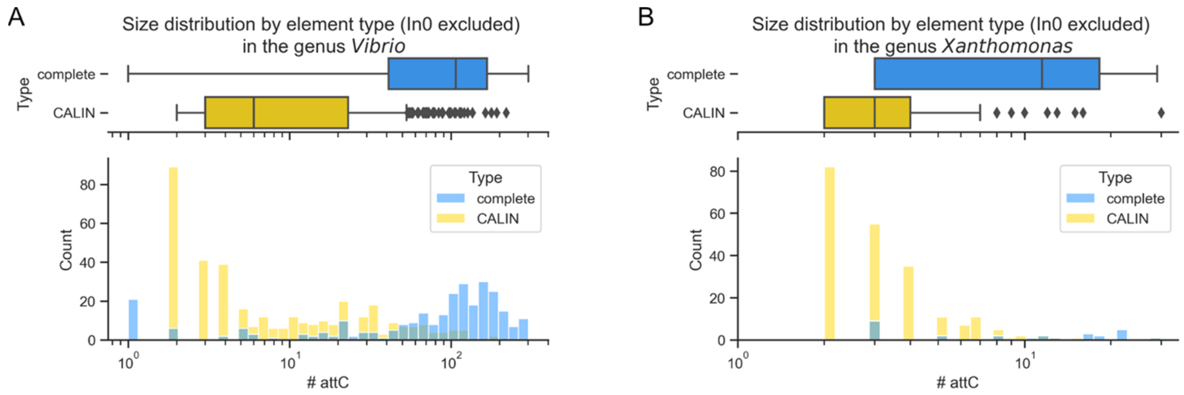

Figure S1. Statistics concerning the distribution of integrons in two genera known for harboring sedentary integrons in RefSeq NCBI database. Distribution of the number of *attC* sites found per element (complete integron or CALIN) in *Vibrio* (**A**) and *Xanthomonas* (**B**).s
